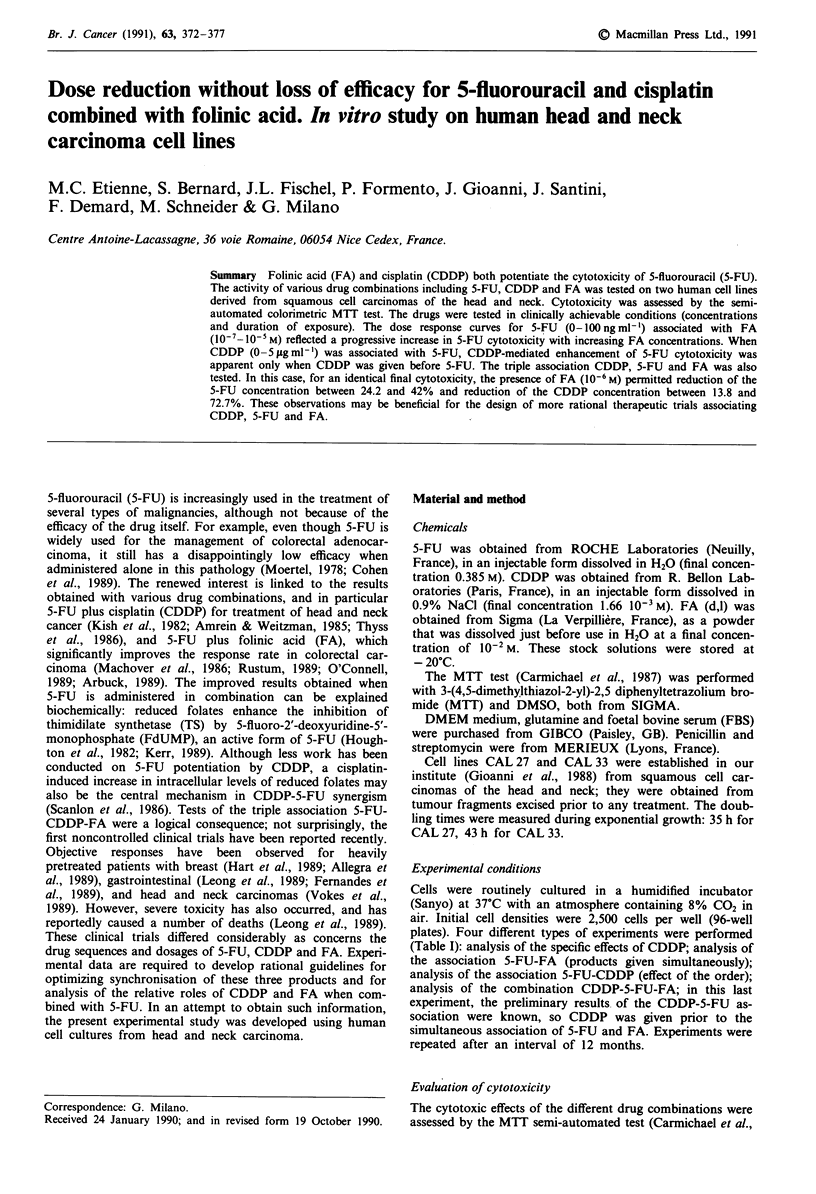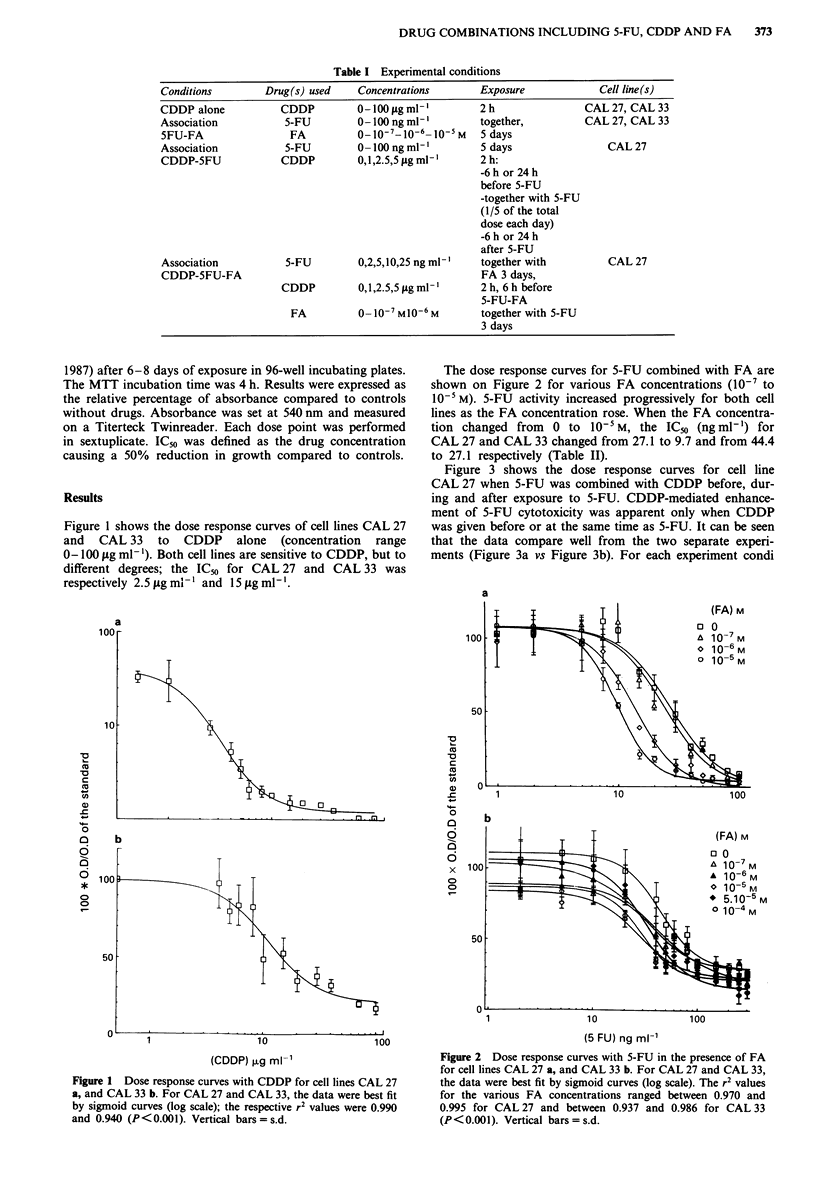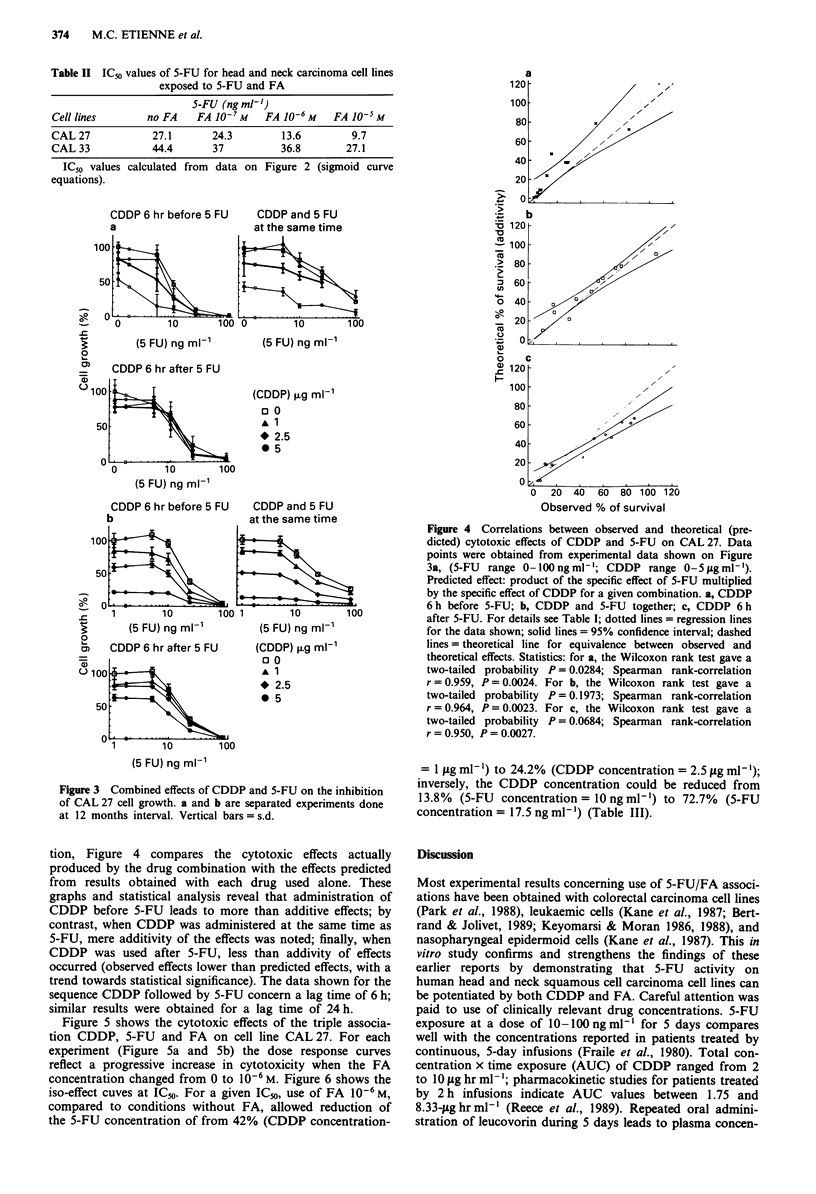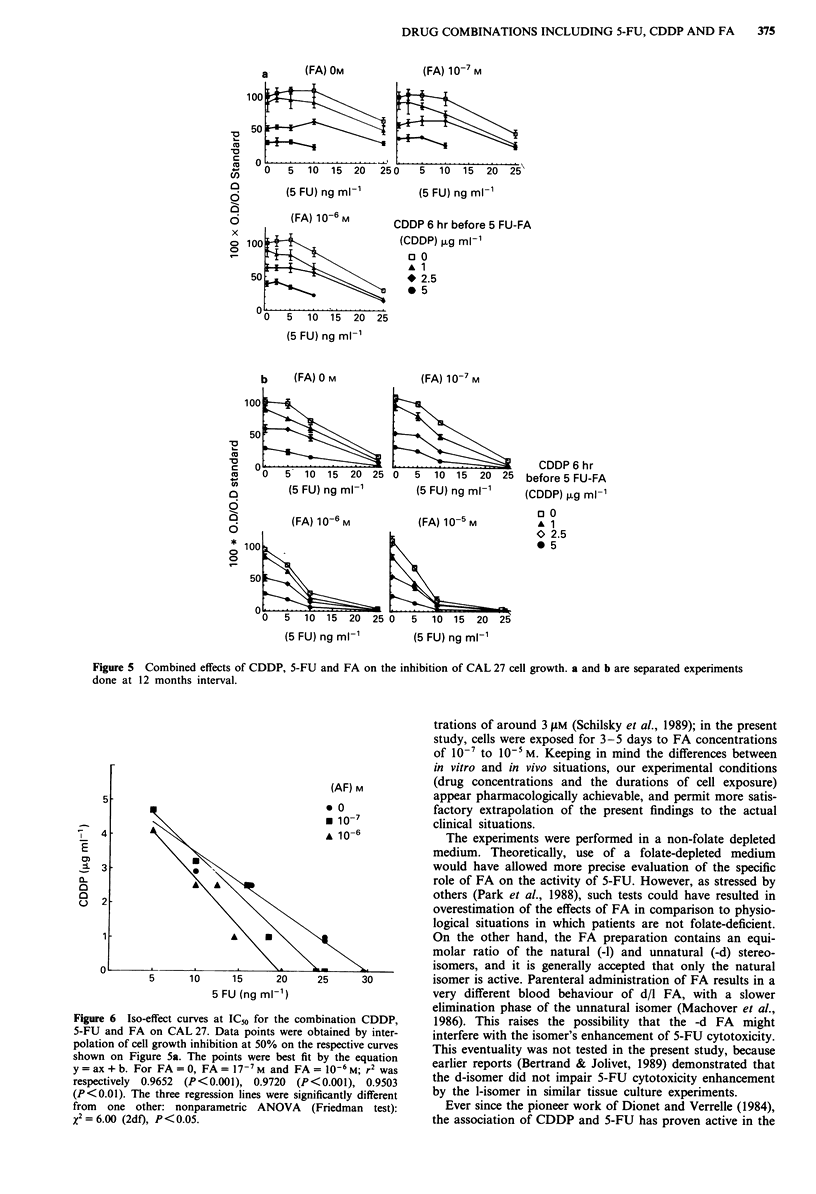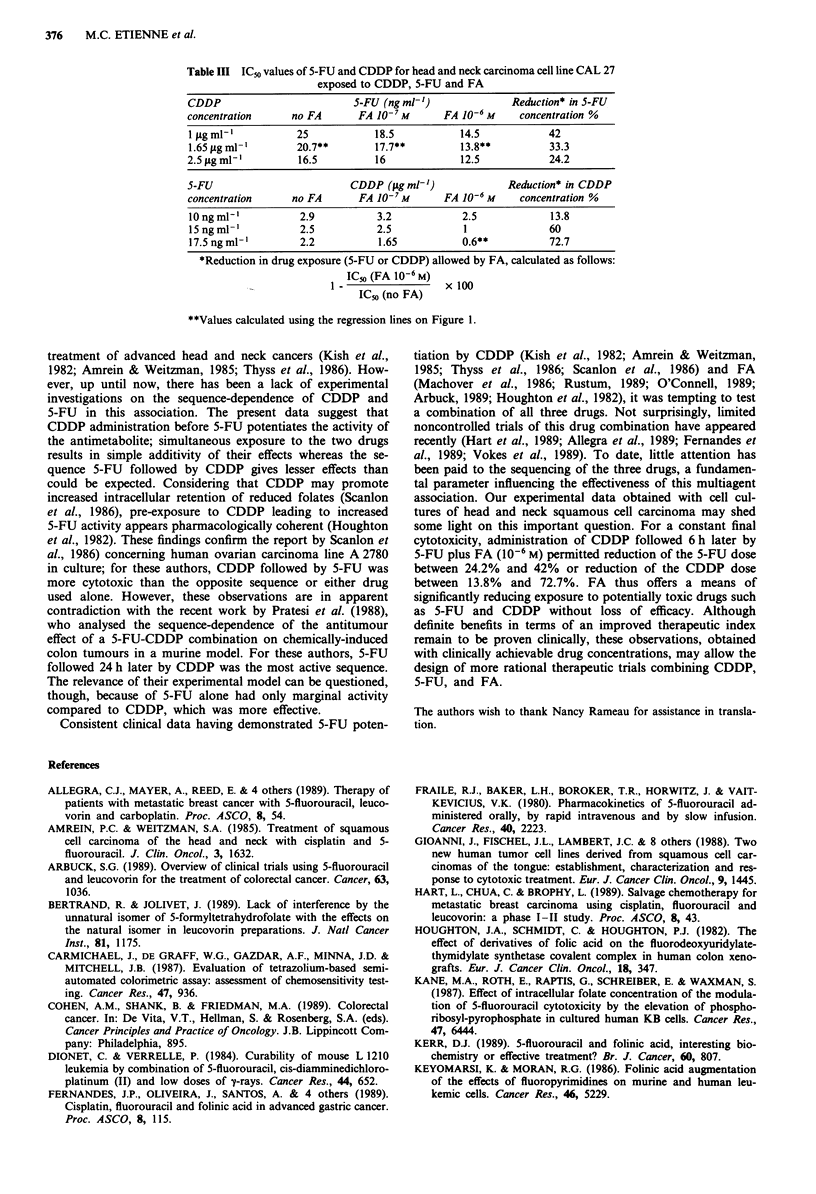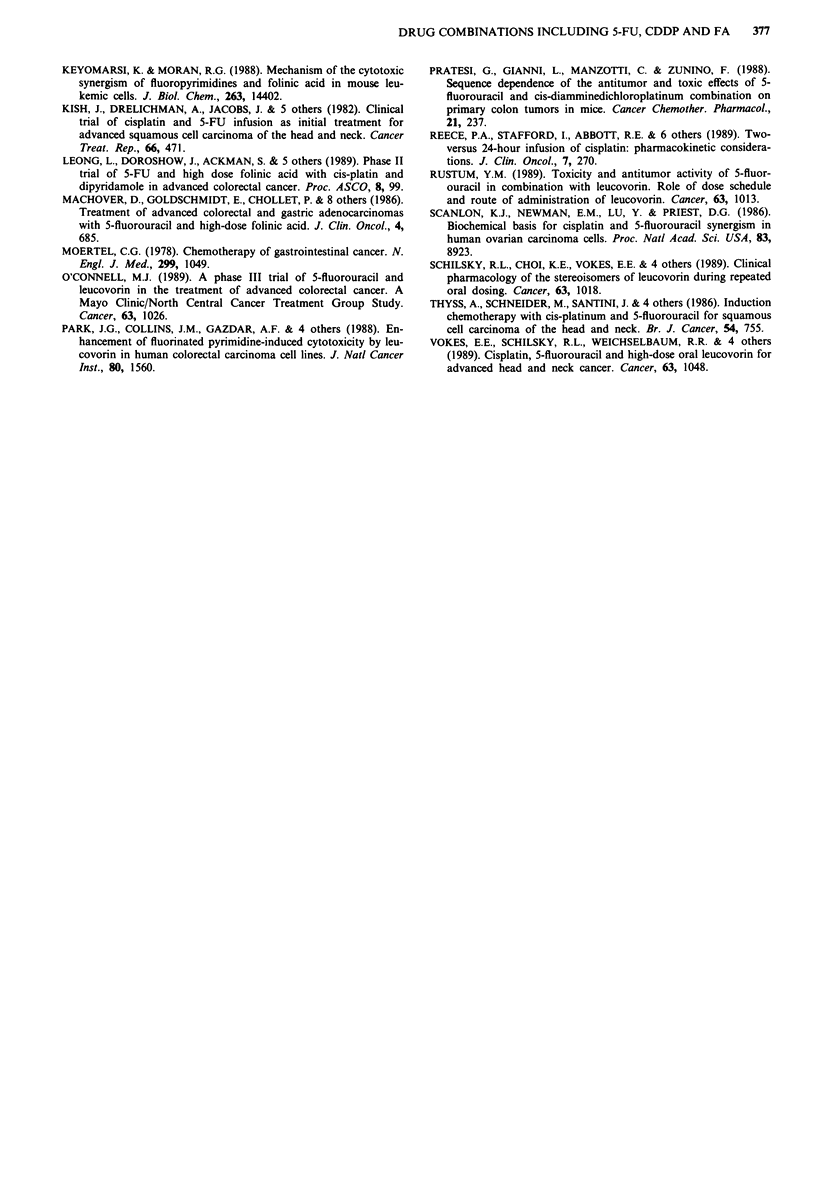# Dose reduction without loss of efficacy for 5-fluorouracil and cisplatin combined with folinic acid. In vitro study on human head and neck carcinoma cell lines.

**DOI:** 10.1038/bjc.1991.88

**Published:** 1991-03

**Authors:** M. C. Etienne, S. Bernard, J. L. Fischel, P. Formento, J. Gioanni, J. Santini, F. Demard, M. Schneider, G. Milano

**Affiliations:** Centre Antoine-Lacassagne, Nice, France.

## Abstract

Folinic acid (FA) and cisplatin (CDDP) both potentiate the cytotoxicity of 5-fluorouracil (5-FU). The activity of various drug combinations including 5-FU, CDDP and FA was tested on two human cell lines derived from squamous cell carcinomas of the head and neck. Cytotoxicity was assessed by the semi-automated colorimetric MTT test. The drugs were tested in clinically achievable conditions (concentrations and duration of exposure). The dose response curves for 5-FU (0-100 ng ml-1) associated with FA (10(-7)-10(-5) M) reflected a progressive increase in 5-FU cytotoxicity with increasing FA concentrations. When CDDP (0-5 micrograms ml-1) was associated with 5-FU, CDDP-mediated enhancement of 5-FU cytotoxicity was apparent only when CDDP was given before 5-FU. The triple association CDDP, 5-FU and FA was also tested. In this case, for an identical final cytotoxicity, the presence of FA (10(-6) M) permitted reduction of the 5-FU concentration between 24.2 and 42% and reduction of the CDDP concentration between 13.8 and 72.7%. These observations may be beneficial for the design of more rational therapeutic trials associating CDDP, 5-FU and FA.


Br. J. Cancer (1991), 63, 372-377 © Macmillan Press Ltd., 1991

### Dose reduction without loss of efficacy for 5-fluorouracil and cisplatin combined with folinic acid. *In vitro* study on human head and neck carcinoma cell lines

M.C. Etienne, S. Bernard, J.L. Fischel, P. Formento, J. Gioanni, J. Santini, F. Demard, M. Schneider & G. Milano

*Centre Antoine-Lacassagne, 36 voie Romaine, 06054 Nice Cedex, France.*

**Summary** Folinic acid (FA) and cisplatin (CDDP) both potentiate the cytotoxicity of 5-fluorouracil (5-FU). The activity of various drug combinations including 5-FU, CDDP and FA was tested on two human cell lines derived from squamous cell carcinomas of the head and neck. Cytotoxicity was assessed by the semi-automated colorimetric MTT test. The drugs were tested in clinically achievable conditions (concentrations and duration of exposure). The dose response curves for 5-FU (0–100 ng $ml^{-1}$) associated with FA ($10^{-7}$–$10^{-5}$ M) reflected a progressive increase in 5-FU cytotoxicity with increasing FA concentrations. When CDDP (0–5 $\mu g\ ml^{-1}$) was associated with 5-FU, CDDP-mediated enhancement of 5-FU cytotoxicity was apparent only when CDDP was given before 5-FU. The triple association CDDP, 5-FU and FA was also tested. In this case, for an identical final cytotoxicity, the presence of FA ($10^{-6}$ M) permitted reduction of the 5-FU concentration between 24.2 and 42% and reduction of the CDDP concentration between 13.8 and 72.7%. These observations may be beneficial for the design of more rational therapeutic trials associating CDDP, 5-FU and FA.

5-fluorouracil (5-FU) is increasingly used in the treatment of several types of malignancies, although not because of the efficacy of the drug itself. For example, even though 5-FU is widely used for the management of colorectal adenocarcinoma, it still has a disappointingly low efficacy when administered alone in this pathology (Moertel, 1978; Cohen *et al.*, 1989). The renewed interest is linked to the results obtained with various drug combinations, and in particular 5-FU plus cisplatin (CDDP) for treatment of head and neck cancer (Kish *et al.*, 1982; Amrein & Weitzman, 1985; Thyss *et al.*, 1986), and 5-FU plus folinic acid (FA), which significantly improves the response rate in colorectal carcinoma (Machover *et al.*, 1986; Rustum, 1989; O'Connell, 1989; Arbuck, 1989). The improved results obtained when 5-FU is administered in combination can be explained biochemically: reduced folates enhance the inhibition of thimidilate synthetase (TS) by 5-fluoro-2'-deoxyuridine-5'-monophosphate (FdUMP), an active form of 5-FU (Houghton *et al.*, 1982; Kerr, 1989). Although less work has been conducted on 5-FU potentiation by CDDP, a cisplatin-induced increase in intracellular levels of reduced folates may also be the central mechanism in CDDP-5-FU synergism (Scanlon *et al.*, 1986). Tests of the triple association 5-FU-CDDP-FA were a logical consequence; not surprisingly, the first noncontrolled clinical trials have been reported recently. Objective responses have been observed for heavily pretreated patients with breast (Hart *et al.*, 1989; Allegra *et al.*, 1989), gastrointestinal (Leong *et al.*, 1989; Fernandes *et al.*, 1989), and head and neck carcinomas (Vokes *et al.*, 1989). However, severe toxicity has also occurred, and has reportedly caused a number of deaths (Leong *et al.*, 1989). These clinical trials differed considerably as concerns the drug sequences and dosages of 5-FU, CDDP and FA. Experimental data are required to develop rational guidelines for optimizing synchronisation of these three products and for analysis of the relative roles of CDDP and FA when combined with 5-FU. In an attempt to obtain such information, the present experimental study was developed using human cell cultures from head and neck carcinoma.

Correspondence: G. Milano.
Received 24 January 1990; and in revised form 19 October 1990.

### Material and method

### *Chemicals*

5-FU was obtained from ROCHE Laboratories (Neuilly, France), in an injectable form dissolved in $H_2O$ (final concentration 0.385 M). CDDP was obtained from R. Bellon Laboratories (Paris, France), in an injectable form dissolved in 0.9% NaCl (final concentration 1.66 $10^{-3}$ M). FA (d,l) was obtained from Sigma (La Verpillière, France), as a powder that was dissolved just before use in $H_2O$ at a final concentration of $10^{-2}$ M. These stock solutions were stored at $-20$°C.

The MTT test (Carmichael *et al.*, 1987) was performed with 3-(4,5-dimethylthiazol-2-yl)-2,5 diphenyltetrazolium bromide (MTT) and DMSO, both from SIGMA.

DMEM medium, glutamine and foetal bovine serum (FBS) were purchased from GIBCO (Paisley, GB). Penicillin and streptomycin were from MERIEUX (Lyons, France).

Cell lines CAL 27 and CAL 33 were established in our institute (Gioanni *et al.*, 1988) from squamous cell carcinomas of the head and neck; they were obtained from tumour fragments excised prior to any treatment. The doubling times were measured during exponential growth: 35 h for CAL 27, 43 h for CAL 33.

### *Experimental conditions*

Cells were routinely cultured in a humidified incubator (Sanyo) at 37°C with an atmosphere containing 8% $CO_2$ in air. Initial cell densities were 2,500 cells per well (96-well plates). Four different types of experiments were performed (Table I): analysis of the specific effects of CDDP; analysis of the association 5-FU-FA (products given simultaneously); analysis of the association 5-FU-CDDP (effect of the order); analysis of the combination CDDP-5-FU-FA; in this last experiment, the preliminary results of the CDDP-5-FU association were known, so CDDP was given prior to the simultaneous association of 5-FU and FA. Experiments were repeated after an interval of 12 months.

### *Evaluation of cytotoxicity*

The cytotoxic effects of the different drug combinations were assessed by the MTT semi-automated test (Carmichael *et al.*,

**Table I** Experimental conditions

| *Conditions* | *Drug(s) used* | *Concentrations* | *Exposure* | *Cell line(s)* |
|---|---|---|---|---|
| CDDP alone | CDDP | 0–100 µg ml$^{-1}$ | 2 h | CAL 27, CAL 33 |
| Association | 5-FU | 0–100 ng ml$^{-1}$ | together, | CAL 27, CAL 33 |
| 5FU-FA | FA | 0–$10^{-7}$–$10^{-6}$–$10^{-5}$ M | 5 days | |
| Association | 5-FU | 0–100 ng ml$^{-1}$ | 5 days | CAL 27 |
| CDDP-5FU | CDDP | 0,1,2.5,5 µg ml$^{-1}$ | 2 h: -6 h or 24 h before 5-FU -together with 5-FU (1/5 of the total dose each day) -6 h or 24 h after 5-FU | |
| Association CDDP-5FU-FA | 5-FU | 0,2,5,10,25 ng ml$^{-1}$ | together with FA 3 days, | CAL 27 |
| | CDDP | 0,1,2.5,5 µg ml$^{-1}$ | 2 h, 6 h before 5-FU-FA | |
| | FA | 0–$10^{-7}$ M$10^{-6}$ M | together with 5-FU 3 days | |

1987) after 6–8 days of exposure in 96-well incubating plates. The MTT incubation time was 4 h. Results were expressed as the relative percentage of absorbance compared to controls without drugs. Absorbance was set at 540 nm and measured on a Titerteck Twinreader. Each dose point was performed in sextuplicate. $IC_{50}$ was defined as the drug concentration causing a 50% reduction in growth compared to controls.

### Results

Figure 1 shows the dose response curves of cell lines CAL 27 and CAL 33 to CDDP alone (concentration range 0–100 µg ml$^{-1}$). Both cell lines are sensitive to CDDP, but to different degrees; the $IC_{50}$ for CAL 27 and CAL 33 was respectively 2.5 µg ml$^{-1}$ and 15 µg ml$^{-1}$.

**Figure 1** Dose response curves with CDDP for cell lines CAL 27 **a**, and CAL 33 **b**. For CAL 27 and CAL 33, the data were best fit by sigmoid curves (log scale); the respective $r^2$ values were 0.990 and 0.940 ($P<0.001$). Vertical bars = s.d.

The dose response curves for 5-FU combined with FA are shown on Figure 2 for various FA concentrations ($10^{-7}$ to $10^{-5}$ M). 5-FU activity increased progressively for both cell lines as the FA concentration rose. When the FA concentration changed from 0 to $10^{-5}$ M, the $IC_{50}$ (ng ml$^{-1}$) for CAL 27 and CAL 33 changed from 27.1 to 9.7 and from 44.4 to 27.1 respectively (Table II).

Figure 3 shows the dose response curves for cell line CAL 27 when 5-FU was combined with CDDP before, during and after exposure to 5-FU. CDDP-mediated enhancement of 5-FU cytotoxicity was apparent only when CDDP was given before or at the same time as 5-FU. It can be seen that the data compare well from the two separate experiments (Figure 3a *vs* Figure 3b). For each experiment condi

**Figure 2** Dose response curves with 5-FU in the presence of FA for cell lines CAL 27 **a**, and CAL 33 **b**. For CAL 27 and CAL 33, the data were best fit by sigmoid curves (log scale). The $r^2$ values for the various FA concentrations ranged between 0.970 and 0.995 for CAL 27 and between 0.937 and 0.986 for CAL 33 ($P<0.001$). Vertical bars = s.d.

**Table II** $IC_{50}$ values of 5-FU for head and neck carcinoma cell lines exposed to 5-FU and FA

| Cell lines | 5-FU ($ng\ ml^{-1}$) no FA | FA $10^{-7}$ M | FA $10^{-6}$ M | FA $10^{-5}$ M |
|---|---|---|---|---|
| CAL 27 | 27.1 | 24.3 | 13.6 | 9.7 |
| CAL 33 | 44.4 | 37 | 36.8 | 27.1 |

$IC_{50}$ values calculated from data on Figure 2 (sigmoid curve equations).

**Figure 3** Combined effects of CDDP and 5-FU on the inhibition of CAL 27 cell growth. **a** and **b** are separated experiments done at 12 months interval. Vertical bars = s.d.

**Figure 4** Correlations between observed and theoretical (predicted) cytotoxic effects of CDDP and 5-FU on CAL 27. Data points were obtained from experimental data shown on Figure 3a, (5-FU range 0–100 ng $ml^{-1}$; CDDP range 0–5 μg $ml^{-1}$). Predicted effect: product of the specific effect of 5-FU multiplied by the specific effect of CDDP for a given combination. **a**, CDDP 6 h before 5-FU; **b**, CDDP and 5-FU together; **c**, CDDP 6 h after 5-FU. For details see Table I; dotted lines = regression lines for the data shown; solid lines = 95% confidence interval; dashed lines = theoretical line for equivalence between observed and theoretical effects. Statistics: for **a**, the Wilcoxon rank test gave a two-tailed probability $P = 0.0284$; Spearman rank-correlation $r = 0.959$, $P = 0.0024$. For **b**, the Wilcoxon rank test gave a two-tailed probability $P = 0.1973$; Spearman rank-correlation $r = 0.964$, $P = 0.0023$. For **c**, the Wilcoxon rank test gave a two-tailed probability $P = 0.0684$; Spearman rank-correlation $r = 0.950$, $P = 0.0027$.

tion, Figure 4 compares the cytotoxic effects actually produced by the drug combination with the effects predicted from results obtained with each drug used alone. These graphs and statistical analysis reveal that administration of CDDP before 5-FU leads to more than additive effects; by contrast, when CDDP was administered at the same time as 5-FU, mere additivity of the effects was noted; finally, when CDDP was used after 5-FU, less than addivity of effects occurred (observed effects lower than predicted effects, with a trend towards statistical significance). The data shown for the sequence CDDP followed by 5-FU concern a lag time of 6 h; similar results were obtained for a lag time of 24 h.

Figure 5 shows the cytotoxic effects of the triple association CDDP, 5-FU and FA on cell line CAL 27. For each experiment (Figure 5a and 5b) the dose response curves reflect a progressive increase in cytotoxicity when the FA concentration changed from 0 to $10^{-6}$ M. Figure 6 shows the iso-effect cuves at $IC_{50}$. For a given $IC_{50}$, use of FA $10^{-6}$ M, compared to conditions without FA, allowed reduction of the 5-FU concentration of from 42% (CDDP concentration = 1 μg $ml^{-1}$) to 24.2% (CDDP concentration = 2.5 μg $ml^{-1}$); inversely, the CDDP concentration could be reduced from 13.8% (5-FU concentration = 10 ng $ml^{-1}$) to 72.7% (5-FU concentration = 17.5 ng $ml^{-1}$) (Table III).

### Discussion

Most experimental results concerning use of 5-FU/FA associations have been obtained with colorectal carcinoma cell lines (Park *et al.*, 1988), leukaemic cells (Kane *et al.*, 1987; Bertrand & Jolivet, 1989; Keyomarsi & Moran 1986, 1988), and nasopharyngeal epidermoid cells (Kane *et al.*, 1987). This *in vitro* study confirms and strengthens the findings of these earlier reports by demonstrating that 5-FU activity on human head and neck squamous cell carcinoma cell lines can be potentiated by both CDDP and FA. Careful attention was paid to use of clinically relevant drug concentrations. 5-FU exposure at a dose of 10–100 ng $ml^{-1}$ for 5 days compares well with the concentrations reported in patients treated by continuous, 5-day infusions (Fraile *et al.*, 1980). Total concentration × time exposure (AUC) of CDDP ranged from 2 to 10 μg hr $ml^{-1}$; pharmacokinetic studies for patients treated by 2 h infusions indicate AUC values between 1.75 and 8.33-μg hr $ml^{-1}$ (Reece *et al.*, 1989). Repeated oral administration of leucovorin during 5 days leads to plasma concen-

**Figure 5** Combined effects of CDDP, 5-FU and FA on the inhibition of CAL 27 cell growth. **a** and **b** are separated experiments done at 12 months interval.

**Figure 6** Iso-effect curves at $IC_{50}$ for the combination CDDP, 5-FU and FA on CAL 27. Data points were obtained by interpolation of cell growth inhibition at 50% on the respective curves shown on Figure 5**a**. The points were best fit by the equation $y = ax + b$. For $FA = 0$, $FA = 17^{-7}$ M and $FA = 10^{-6}$ M; $r^2$ was respectively 0.9652 ($P < 0.001$), 0.9720 ($P < 0.001$), 0.9503 ($P < 0.01$). The three regression lines were significantly different from one other: nonparametric ANOVA (Friedman test): $\chi^2 = 6.00$ (2df), $P < 0.05$.

trations of around 3 μM (Schilsky *et al.*, 1989); in the present study, cells were exposed for 3–5 days to FA concentrations of $10^{-7}$ to $10^{-5}$ M. Keeping in mind the differences between *in vitro* and *in vivo* situations, our experimental conditions (drug concentrations and the durations of cell exposure) appear pharmacologically achievable, and permit more satisfactory extrapolation of the present findings to the actual clinical situations.

The experiments were performed in a non-folate depleted medium. Theoretically, use of a folate-depleted medium would have allowed more precise evaluation of the specific role of FA on the activity of 5-FU. However, as stressed by others (Park *et al.*, 1988), such tests could have resulted in overestimation of the effects of FA in comparison to physiological situations in which patients are not folate-deficient. On the other hand, the FA preparation contains an equimolar ratio of the natural (-l) and unnatural (-d) stereoisomers, and it is generally accepted that only the natural isomer is active. Parenteral administration of FA results in a very different blood behaviour of d/l FA, with a slower elimination phase of the unnatural isomer (Machover *et al.*, 1986). This raises the possibility that the -d FA might interfere with the isomer's enhancement of 5-FU cytotoxicity. This eventuality was not tested in the present study, because earlier reports (Bertrand & Jolivet, 1989) demonstrated that the d-isomer did not impair 5-FU cytotoxicity enhancement by the l-isomer in similar tissue culture experiments.

Ever since the pioneer work of Dionet and Verrelle (1984), the association of CDDP and 5-FU has proven active in the

**Table III** $IC_{50}$ values of 5-FU and CDDP for head and neck carcinoma cell line CAL 27 exposed to CDDP, 5-FU and FA

| *CDDP concentration* | *no FA* | *5-FU (ng ml⁻¹)* *FA $10^{-7}$ M* | *FA $10^{-6}$ M* | *Reduction* in 5-FU concentration %* |
|---|---|---|---|---|
| 1 μg ml$^{-1}$ | 25 | 18.5 | 14.5 | 42 |
| 1.65 μg ml$^{-1}$ | 20.7** | 17.7** | 13.8** | 33.3 |
| 2.5 μg ml$^{-1}$ | 16.5 | 16 | 12.5 | 24.2 |

| *5-FU concentration* | *no FA* | *CDDP (μg ml⁻¹)* *FA $10^{-7}$ M* | *FA $10^{-6}$ M* | *Reduction* in CDDP concentration %* |
|---|---|---|---|---|
| 10 ng ml$^{-1}$ | 2.9 | 3.2 | 2.5 | 13.8 |
| 15 ng ml$^{-1}$ | 2.5 | 2.5 | 1 | 60 |
| 17.5 ng ml$^{-1}$ | 2.2 | 1.65 | 0.6** | 72.7 |

*Reduction in drug exposure (5-FU or CDDP) allowed by FA, calculated as follows:

$$1 - \frac{IC_{50}\ (FA\ 10^{-6}\ M)}{IC_{50}\ (no\ FA)} \times 100$$

**Values calculated using the regression lines on Figure 1.

treatment of advanced head and neck cancers (Kish *et al.*, 1982; Amrein & Weitzman, 1985; Thyss *et al.*, 1986). However, up until now, there has been a lack of experimental investigations on the sequence-dependence of CDDP and 5-FU in this association. The present data suggest that CDDP administration before 5-FU potentiates the activity of the antimetabolite; simultaneous exposure to the two drugs results in simple additivity of their effects whereas the sequence 5-FU followed by CDDP gives lesser effects than could be expected. Considering that CDDP may promote increased intracellular retention of reduced folates (Scanlon *et al.*, 1986), pre-exposure to CDDP leading to increased 5-FU activity appears pharmacologically coherent (Houghton *et al.*, 1982). These findings confirm the report by Scanlon *et al.*, 1986) concerning human ovarian carcinoma line A 2780 in culture; for these authors, CDDP followed by 5-FU was more cytotoxic than the opposite sequence or either drug used alone. However, these observations are in apparent contradiction with the recent work by Pratesi *et al.* (1988), who analysed the sequence-dependence of the antitumour effect of a 5-FU-CDDP combination on chemically-induced colon tumours in a murine model. For these authors, 5-FU followed 24 h later by CDDP was the most active sequence. The relevance of their experimental model can be questioned, though, because of 5-FU alone had only marginal activity compared to CDDP, which was more effective.

Consistent clinical data having demonstrated 5-FU potentiation by CDDP (Kish *et al.*, 1982; Amrein & Weitzman, 1985; Thyss *et al.*, 1986; Scanlon *et al.*, 1986) and FA (Machover *et al.*, 1986; Rustum, 1989; O'Connell, 1989; Arbuck, 1989; Houghton *et al.*, 1982), it was tempting to test a combination of all three drugs. Not surprisingly, limited noncontrolled trials of this drug combination have appeared recently (Hart *et al.*, 1989; Allegra *et al.*, 1989; Fernandes *et al.*, 1989; Vokes *et al.*, 1989). To date, little attention has been paid to the sequencing of the three drugs, a fundamental parameter influencing the effectiveness of this multiagent association. Our experimental data obtained with cell cultures of head and neck squamous cell carcinoma may shed some light on this important question. For a constant final cytotoxicity, administration of CDDP followed 6 h later by 5-FU plus FA ($10^{-6}$ M) permitted reduction of the 5-FU dose between 24.2% and 42% or reduction of the CDDP dose between 13.8% and 72.7%. FA thus offers a means of significantly reducing exposure to potentially toxic drugs such as 5-FU and CDDP without loss of efficacy. Although definite benefits in terms of an improved therapeutic index remain to be proven clinically, these observations, obtained with clinically achievable drug concentrations, may allow the design of more rational therapeutic trials combining CDDP, 5-FU, and FA.

The authors wish to thank Nancy Rameau for assistance in translation.

### References

ALLEGRA, C.J., MAYER, A., REED, E. & 4 others (1989). Therapy of patients with metastatic breast cancer with 5-fluorouracil, leucovorin and carboplatin. *Proc. ASCO*, **8**, 54.

AMREIN, P.C. & WEITZMAN, S.A. (1985). Treatment of squamous cell carcinoma of the head and neck with cisplatin and 5-fluorouracil. *J. Clin. Oncol.*, **3**, 1632.

ARBUCK, S.G. (1989). Overview of clinical trials using 5-fluorouracil and leucovorin for the treatment of colorectal cancer. *Cancer*, **63**, 1036.

BERTRAND, R. & JOLIVET, J. (1989). Lack of interference by the unnatural isomer of 5-formyltetrahydrofolate with the effects on the natural isomer in leucovorin preparations. *J. Natl Cancer Inst.*, **81**, 1175.

CARMICHAEL, J., DE GRAFF, W.G., GAZDAR, A.F., MINNA, J.D. & MITCHELL, J.B. (1987). Evaluation of tetrazolium-based semiautomated colorimetric assay: assessment of chemosensitivity testing. *Cancer Res.*, **47**, 936.

COHEN, A.M., SHANK, B. & FRIEDMAN, M.A. (1989). Colorectal cancer. In: De Vita, V.T., Hellman, S. & Rosenberg, S.A. (eds). *Cancer Principles and Practice of Oncology*. J.B. Lippincott Company: Philadelphia, 895.

DIONET, C. & VERRELLE, P. (1984). Curability of mouse L 1210 leukemia by combination of 5-fluorouracil, cis-diamminedichloroplatinum (II) and low doses of γ-rays. *Cancer Res.*, **44**, 652.

FERNANDES, J.P., OLIVEIRA, J., SANTOS, A. & 4 others (1989). Cisplatin, fluorouracil and folinic acid in advanced gastric cancer. *Proc. ASCO*, **8**, 115.

FRAILE, R.J., BAKER, L.H., BOROKER, T.R., HORWITZ, J. & VAITKEVICIUS, V.K. (1980). Pharmacokinetics of 5-fluorouracil administered orally, by rapid intravenous and by slow infusion. *Cancer Res.*, **40**, 2223.

GIOANNI, J., FISCHEL, J.L., LAMBERT, J.C. & 8 others (1988). Two new human tumor cell lines derived from squamous cell carcinomas of the tongue: establishment, characterization and response to cytotoxic treatment. *Eur. J. Cancer Clin. Oncol.*, **9**, 1445.

HART, L., CHUA, C. & BROPHY, L. (1989). Salvage chemotherapy for metastatic breast carcinoma using cisplatin, fluorouracil and leucovorin: a phase I–II study. *Proc. ASCO*, **8**, 43.

HOUGHTON, J.A., SCHMIDT, C. & HOUGHTON, P.J. (1982). The effect of derivatives of folic acid on the fluorodeoxyuridylate-thymidylate synthetase covalent complex in human colon xenografts. *Eur. J. Cancer Clin. Oncol.*, **18**, 347.

KANE, M.A., ROTH, E., RAPTIS, G., SCHREIBER, E. & WAXMAN, S. (1987). Effect of intracellular folate concentration of the modulation of 5-fluorouracil cytotoxicity by the elevation of phosphoribosyl-pyrophosphate in cultured human KB cells. *Cancer Res.*, **47**, 6444.

KERR, D.J. (1989). 5-fluorouracil and folinic acid, interesting biochemistry or effective treatment? *Br. J. Cancer*, **60**, 807.

KEYOMARSI, K. & MORAN, R.G. (1986). Folinic acid augmentation of the effects of fluoropyrimidines on murine and human leukemic cells. *Cancer Res.*, **46**, 5229.

KEYOMARSI, K. & MORAN, R.G. (1988). Mechanism of the cytotoxic synergism of fluoropyrimidines and folinic acid in mouse leukemic cells. *J. Biol. Chem.*, **263**, 14402.

KISH, J., DRELICHMAN, A., JACOBS, J. & 5 others (1982). Clinical trial of cisplatin and 5-FU infusion as initial treatment for advanced squamous cell carcinoma of the head and neck. *Cancer Treat. Rep.*, **66**, 471.

LEONG, L., DOROSHOW, J., ACKMAN, S. & 5 others (1989). Phase II trial of 5-FU and high dose folinic acid with cis-platin and dipyridamole in advanced colorectal cancer. *Proc. ASCO*, **8**, 99.

MACHOVER, D., GOLDSCHMIDT, E., CHOLLET, P. & 8 others (1986). Treatment of advanced colorectal and gastric adenocarcinomas with 5-fluorouracil and high-dose folinic acid. *J. Clin. Oncol.*, **4**, 685.

MOERTEL, C.G. (1978). Chemotherapy of gastrointestinal cancer. *N. Engl. J. Med.*, **299**, 1049.

O'CONNELL, M.J. (1989). A phase III trial of 5-fluorouracil and leucovorin in the treatment of advanced colorectal cancer. A Mayo Clinic/North Central Cancer Treatment Group Study. *Cancer*, **63**, 1026.

PARK, J.G., COLLINS, J.M., GAZDAR, A.F. & 4 others (1988). Enhancement of fluorinated pyrimidine-induced cytotoxicity by leucovorin in human colorectal carcinoma cell lines. *J. Natl Cancer Inst.*, **80**, 1560.

PRATESI, G., GIANNI, L., MANZOTTI, C. & ZUNINO, F. (1988). Sequence dependence of the antitumor and toxic effects of 5-fluorouracil and cis-diamminedichloroplatinum combination on primary colon tumors in mice. *Cancer Chemother. Pharmacol.*, **21**, 237.

REECE, P.A., STAFFORD, I., ABBOTT, R.E. & 6 others (1989). Two- versus 24-hour infusion of cisplatin: pharmacokinetic considerations. *J. Clin. Oncol.*, **7**, 270.

RUSTUM, Y.M. (1989). Toxicity and antitumor activity of 5-fluorouracil in combination with leucovorin. Role of dose schedule and route of administration of leucovorin. *Cancer*, **63**, 1013.

SCANLON, K.J., NEWMAN, E.M., LU, Y. & PRIEST, D.G. (1986). Biochemical basis for cisplatin and 5-fluorouracil synergism in human ovarian carcinoma cells. *Proc. Natl Acad. Sci. USA*, **83**, 8923.

SCHILSKY, R.L., CHOI, K.E., VOKES, E.E. & 4 others (1989). Clinical pharmacology of the stereoisomers of leucovorin during repeated oral dosing. *Cancer*, **63**, 1018.

THYSS, A., SCHNEIDER, M., SANTINI, J. & 4 others (1986). Induction chemotherapy with cis-platinum and 5-fluorouracil for squamous cell carcinoma of the head and neck. *Br. J. Cancer*, **54**, 755.

VOKES, E.E., SCHILSKY, R.L., WEICHSELBAUM, R.R. & 4 others (1989). Cisplatin, 5-fluorouracil and high-dose oral leucovorin for advanced head and neck cancer. *Cancer*, **63**, 1048.